# Supplementary figures and images for: Detection of multiple circulating Leishmania species in Lutzomyia longipalpis in the city of Governador Valadares, southeastern Brazil
Source: PLoS One. 2019 Feb 5;14(2):e0211831. doi: 10.1371/journal.pone.0211831 (PMC6363391; doi:10.1371/journal.pone.0211831)

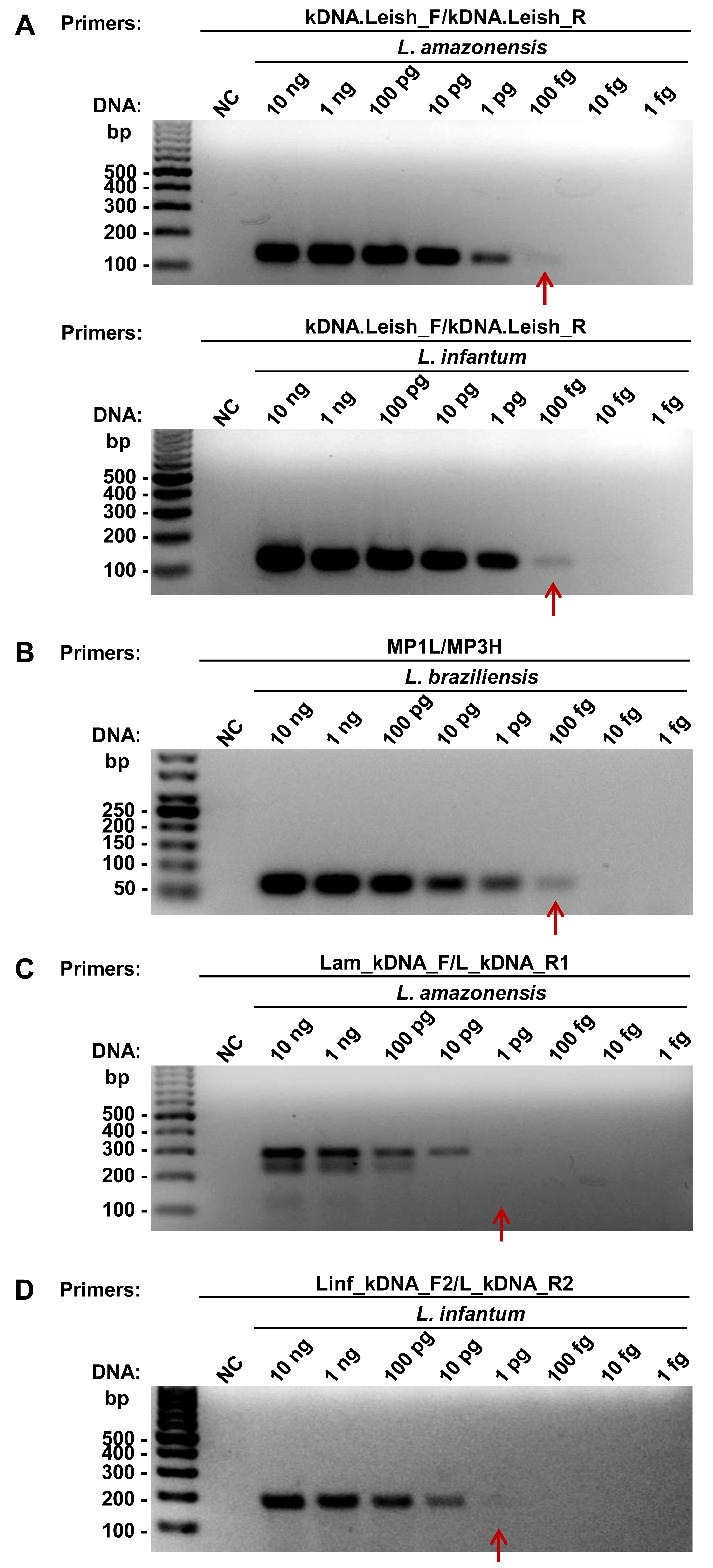

Supplement: S1 Fig — The detection limit of each PCR is indicated by the red arrow: (A) 100 fg of L. amazonensis and L. infantum gDNA for PCR with pair of primer kDNA.Leish; (B) 100 fg of L. braziliensis gDNA for primers MP1L/MP3H; (C) 1 pg of L. amazonensis gDNA for primers Lam_kDNA_F/L_kDNA_R1; and (D) 1 pg of L. infantum gDNA for primers Linf_kDNA_F2/L_kDNA_R2. bp: molecular size marker in base pairs; NC: negative control (without DNA); gDNA: genomic DNA. (TIFF) [file pone.0211831.s001.tiff]

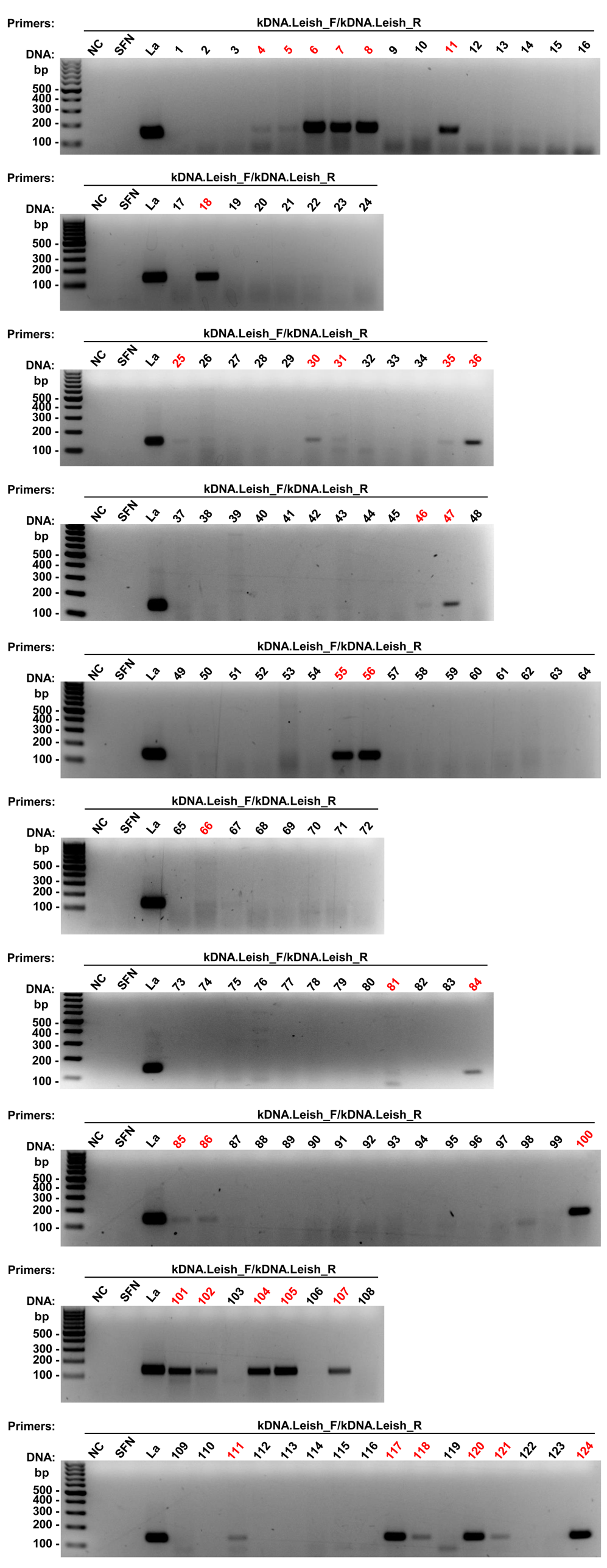

Supplement: S2 Fig — Gels of the PCR with pool gDNA sand flies, using the pair of primers kDNA.Leish, which amplifies Leishmania subgenus with a fragment of 135 bp. Each pool contains 5 DNA samples of Lu. longipalpis, totaling 616 sand flies distributed in 124 pools. Pools marked in red were positive for the primers tested. bp: molecular size marker in base pairs; NC: negative control (without DNA); SFN: not infected sand fly; DNA control: La: L. amazonensis. (TIFF) [file pone.0211831.s002.tiff]

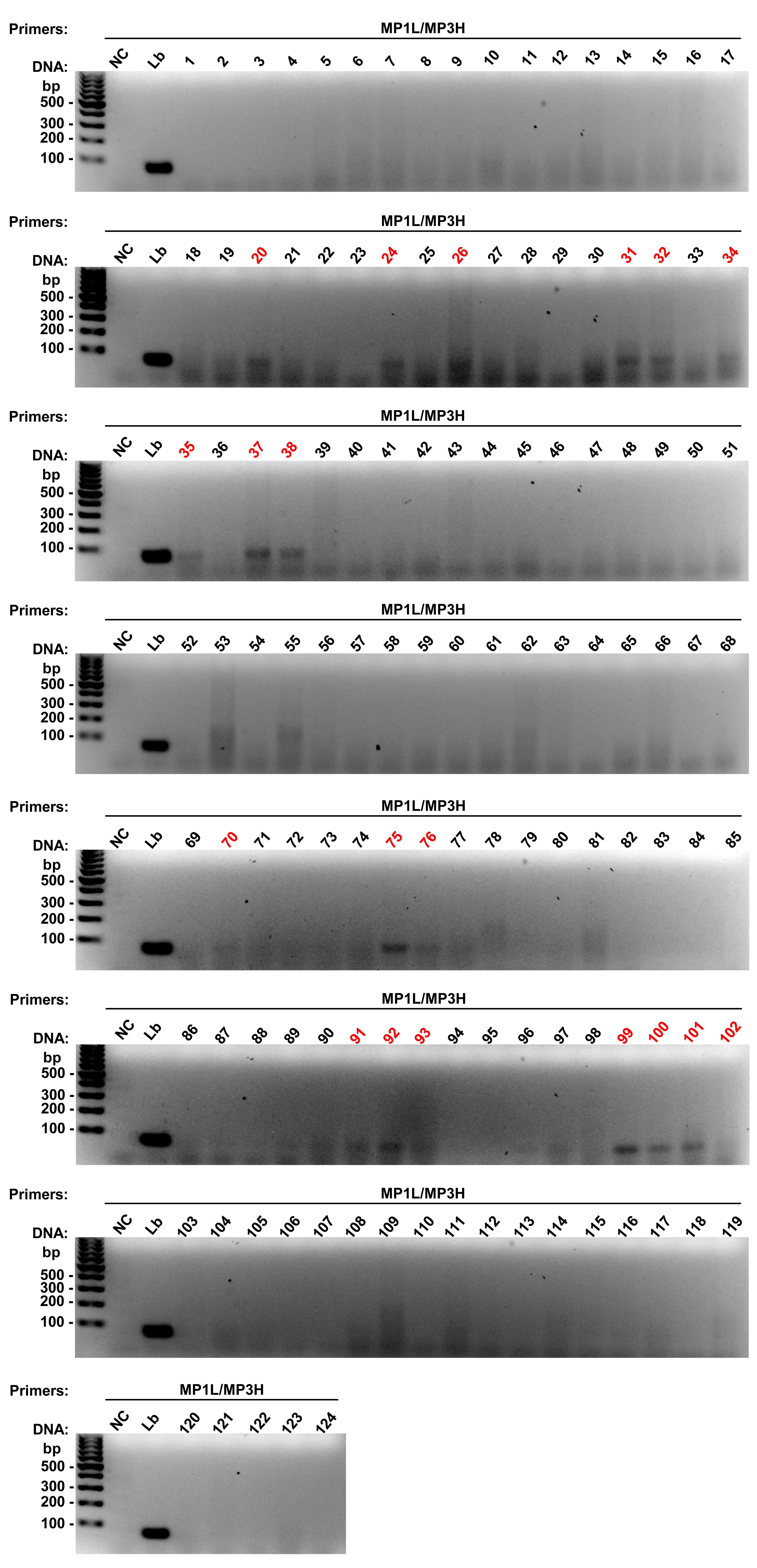

Supplement: S3 Fig — Gels of the PCR with pool gDNA sand flies, using the pair of primers MP1L/MP3H, which amplifies Viannia subgenus with a fragment of 70 bp. Each pool contains 5 DNA samples of Lu. longipalpis, totaling 616 sand flies distributed in 124 pools. Pools marked in red were positive for the primers tested. bp: molecular size marker in base pairs; NC: negative control (without DNA); SFN: not infected sand fly; DNA control: Lb: L. braziliensis. (TIFF) [file pone.0211831.s003.tiff]

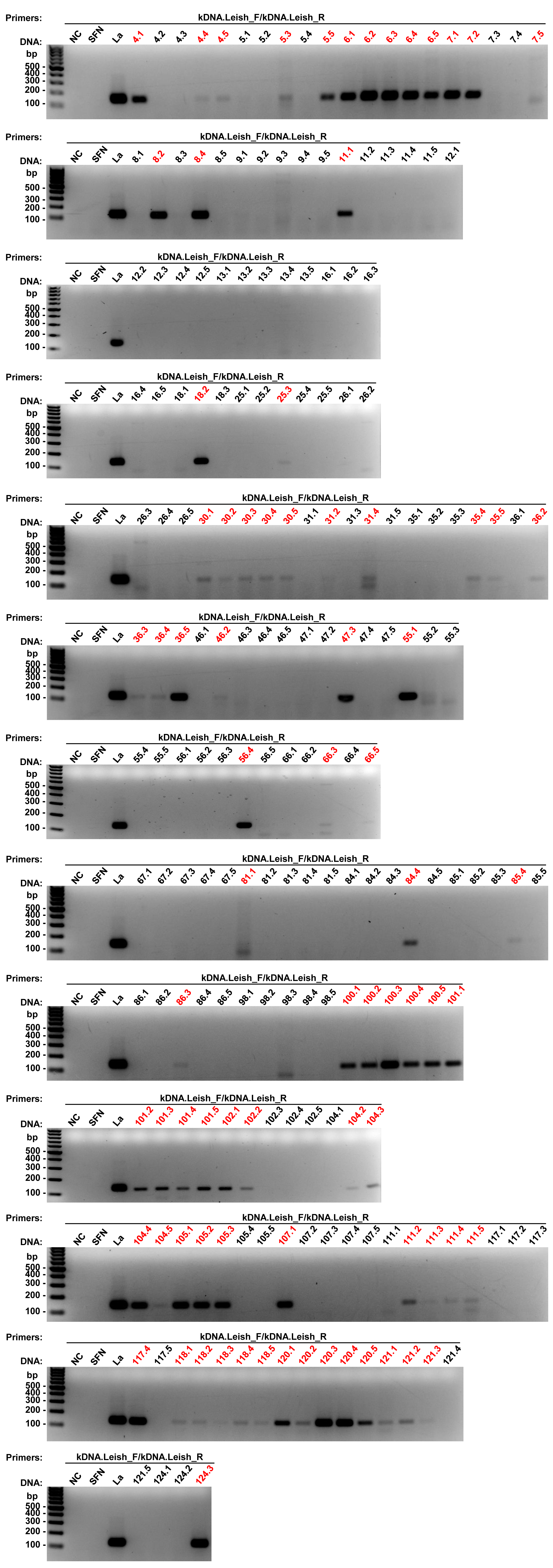

Supplement: S4 Fig — Gels of the PCR with individual DNA samples from positive pools of sand flies, using the pair of primers kDNA.Leish, which amplifies Leishmania subgenus with a fragment of 135 bp. We detected 80 sand flies infected with L. (Leishmania) spp. Samples marked in red were positive for the primers tested. bp: molecular size marker in base pairs; NC: negative control (without DNA); SFN: not infected sand fly; DNA control: La: L. amazonensis. (TIFF) [file pone.0211831.s004.tiff]

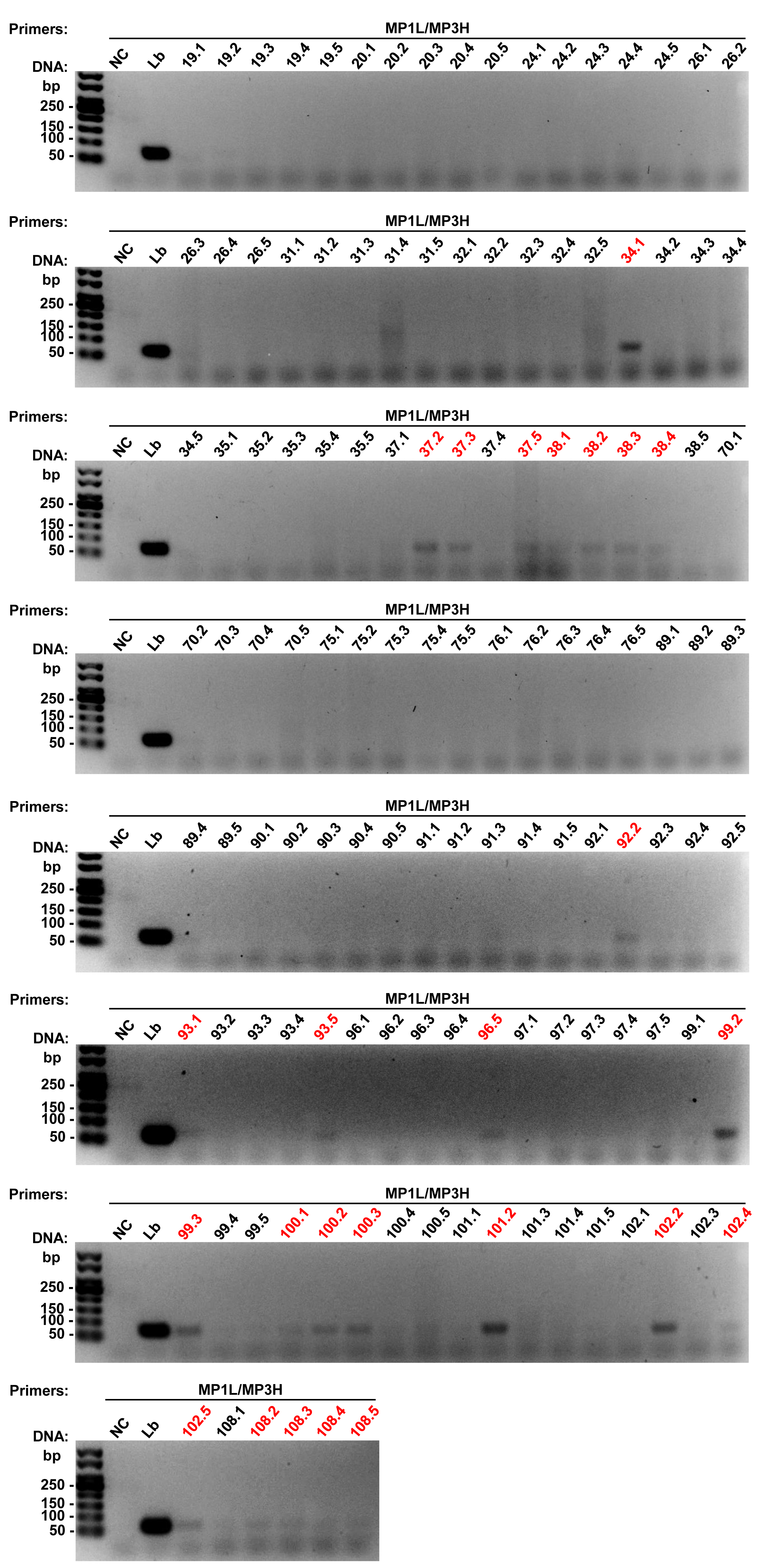

Supplement: S5 Fig — Gels of the PCR with individual DNA samples from positive pools of sand flies, using the pair of primers MP1L/MP3H, which amplifies Viannia subgenus with a fragment of 70 bp. We detected 25 sand flies infected with L. (Viannia) spp. Samples marked in red were positive for the primers tested. bp: molecular size marker in base pairs; NC: negative control (without DNA); SFN: not infected sand fly; DNA control: Lb: L. braziliensis. (TIFF) [file pone.0211831.s005.tiff]

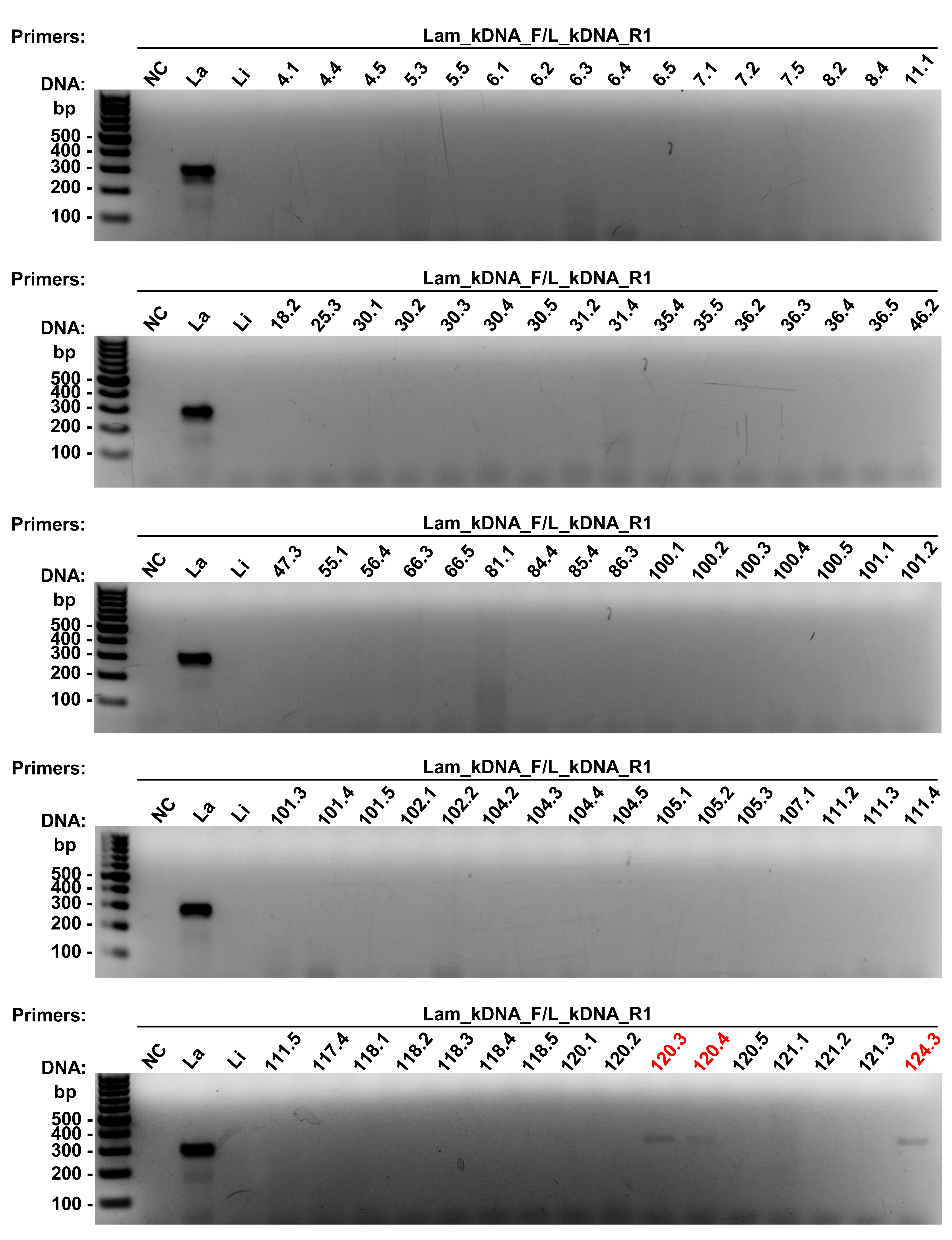

Supplement: S6 Fig — Gels of the PCR with positive DNA samples for Leishmania subgenus, using the pair of primers Lam_kDNA_F/L_kDNA_R1, which amplifies L. amazonensis with a fragment of 294 bp. We detected 3 sand flies infected with L. amazonensis. Samples marked in red were positive for the primers tested. bp: molecular size marker in base pairs; NC: negative control (without DNA); SFN: not infected sand fly; DNA control: La: L. amazonensis; Li: L. infantum. (TIFF) [file pone.0211831.s006.tiff]

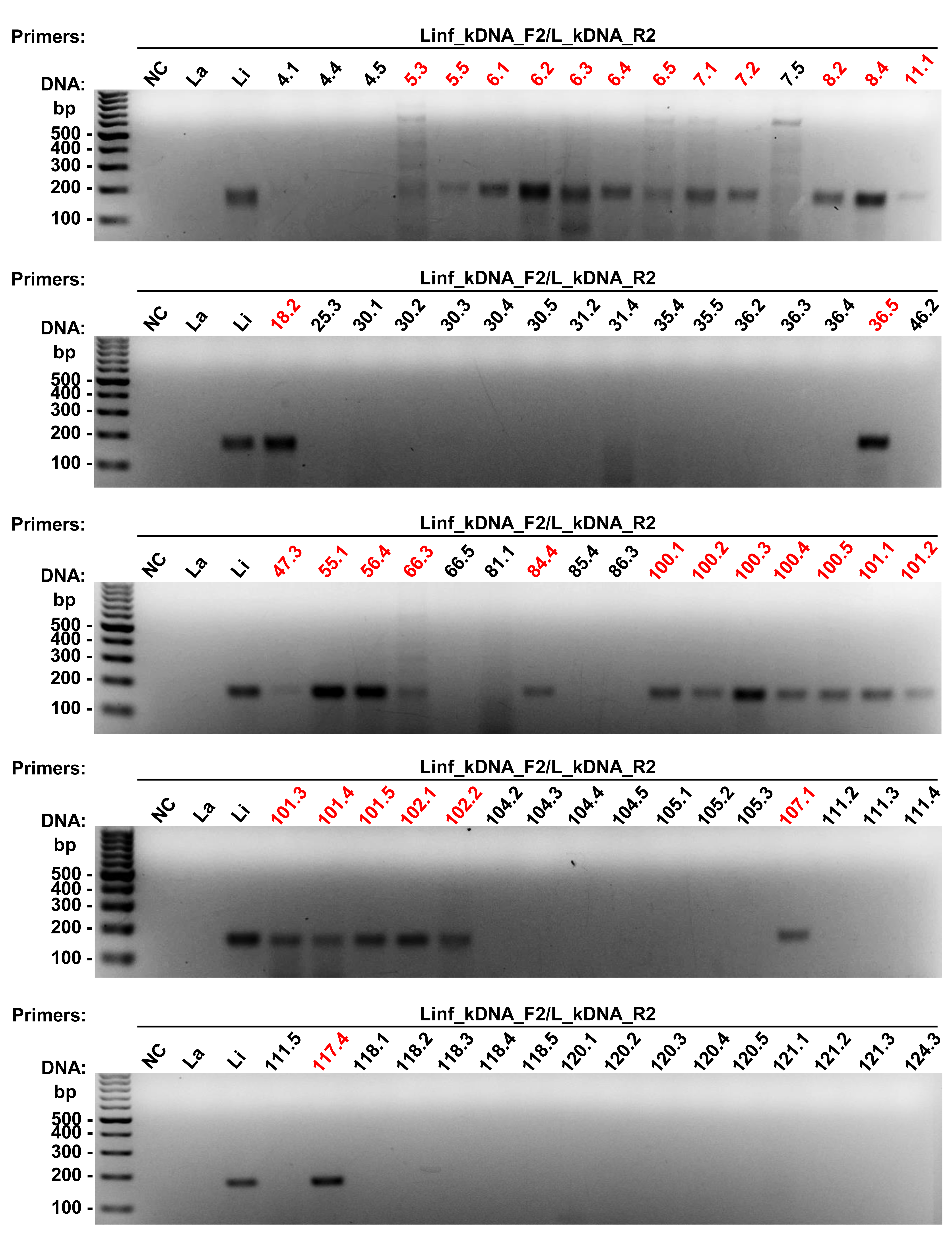

Supplement: S7 Fig — Gels of the PCR with positive DNA samples for Leishmania subgenus, using the pair of primers Linf_kDNA_F2/L_kDNA_R2, which amplifies L. infantum with a fragment of 183 bp. We detected 33 sand flies infected with L. infantum. Samples marked in red were positive for the primers tested. bp: molecular size marker in base pairs; NC: negative control (without DNA); SFN: not infected sand fly; DNA control: La: L. amazonensis; Li: L. infantum. (TIFF) [file pone.0211831.s007.tiff]
